# Pancreatic Neuroendocrine Tumor (PNET) Presenting as a Pseudocyst: A Case Report

**DOI:** 10.7759/cureus.29617

**Published:** 2022-09-26

**Authors:** Swastika Sedhai, Fathia Mohammed, Saveeta Sahtiya, Sadaf Sanaullah, Payal Pritwani, Faraz Saleem, Ayodeji Abere, Muhammad Abu Zar Ghaffari

**Affiliations:** 1 Medicine, Kathmandu University, Kathmandu, NPL; 2 Internal Medicine, University of Gezira, Madani, SDN; 3 Internal Medicine, People's University of Medical & Health Sciences, Nawabshah, Karachi, PAK; 4 Internal Medicine, University of Tripoli Faculty of Medicine, Tripoli, LBY; 5 Internal Medicine, Ghulam Muhammad Mahar Medical College, Sukkur, PAK; 6 Internal Medicine, Akhtar Saeed Medical and Dental College, Lahore, PAK; 7 Internal Medicine, Karazin National University, Kharkov, UKR

**Keywords:** pseudocyst, primitive neuroectodermal tumor (pnet), pnet, pancreas tumor, neuroendocrine, pancreatic neuroendocrine tumor

## Abstract

Pancreatic neuroendocrine tumors (PNETs) account for a very small proportion of all pancreatic tumors. The presence or absence of a specific clinical manifestation associated with hormone oversecretion determines whether a PNET is functional or nonfunctional. Imaging expressions differ significantly, from the common to the extremely rare. Diffuse, uniform pancreatic enlargement, without abnormalities in contour or a central mass, is the most common radiological finding. We report the case of a 43-year-old male who presented with abdominal pain and early satiety over the course of two months and was found to have a non-functioning pancreatic neuroendocrine tumor, with the pseudocyst being the initial diagnostic finding. In comparison to patients with exocrine pancreatic cancer, those with PNET have a much better prognosis and longer expected survival time. This case report highlights the importance of the diagnostic evaluation of PNET and timely intervention to prolong the survival of the patient.

## Introduction

Neuroendocrine tumors (NETs) are epithelial neoplasms characterized by a predominance of neuroendocrine differentiation [1]. A pancreatic neuroendocrine tumor (PNET) is a neuroendocrine tumor (NET) of pancreatic origin. Although PNETs are uncommon tumors, their incidence and frequency have risen significantly [2]. The incidence is between one and two per 100,000 men and women. The majority of these cancers are well-differentiated and progress slowly [3].

PNETs consist of both functional PNETs with clinical symptoms caused by hormones released by the tumors and nonfunctional PNETs with no distinguishable clinical manifestations [1,2]. Due to the common vague clinical presentation, the start of symptoms is typically delayed, and the disease is typically diagnosed at an advanced stage [3]. The selected treatment modality is tumor resection [3]. The purpose of this study was to describe a case of a pancreas neuroendocrine tumor presenting with epigastric pain and initially diagnosed as a pseudocyst.

## Case presentation

A 43-year-old Asian man presented to the outpatient department (OPD) with complaints of epigastric pain and early satiety for the last two months. The pain was sudden in onset, acute in nature, did not radiate to the sides, and could be temporarily alleviated by bending forward. It was not accompanied by nausea or vomiting. No history of weight loss or jaundice was present. The patient is a non-smoker and denied any alcohol intake. The patient is a construction worker and has a body mass index (BMI) of 19.5. He denied experiencing any appetite loss or any chronic illness in the past. His prior medical history was unremarkable.

His vitals were stable: heart rate of 80 beats per minute, blood pressure of 120/80 mmHg, and body temperature of 98 °F. His physical examination was unremarkable except for mild epigastric tenderness on deep palpation. A series of laboratories were conducted (Table 1).

**Table 1:** Lab parameters of the patient MCV: mean corpuscular volume; ALT: alanine transaminase; AST: aspartate transaminase; BUN: blood urea nitrogen; Cr: creatinine; TSH: thyroid-simulating hormone; INR: international normalized ratio; HbA1c: glycated hemoglobin

Lab Parameters	Value	NORMAL RANGE
Hb (g/dL)	10.6	(13.5 - 17.5)
MCV (fl)	86.8	(80 - 100)
WBC (X10^9^/l)	7.2	(4.5 - 11)
Platelets (X10^3^/ul)	330	(150 - 400)
ALT (IU/L)	24	(7 to 55)
AST (IU/L)	33	(8 to 48)
BUN (mg/dL)	24	(6 to 24)
Cr (mg/dL)	0.8	(0.7 to 1.3)
Amylase (IU/L)	110	(30 to 110)
Lipase (IU/L)	70	(10 to 140)
TSH (mU/L)	2.2	(0.4 to 4.0)
INR	1	(1.1 or below)
HbA1c %	5.1	(Below 5.7%)

**Figure 1:**
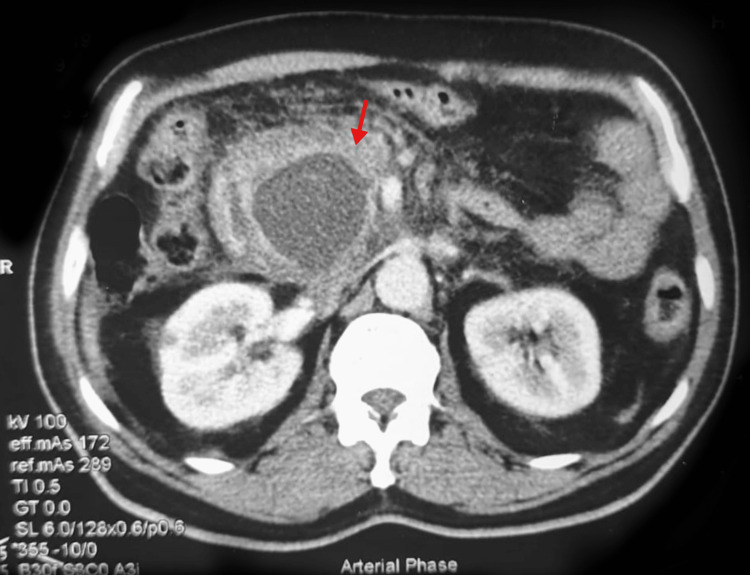
Pseudocyst in the pancreas on CT scan abdomen

His chest X-ray and IgG4 levels were insignificant. The patient was admitted for further evaluation and an abdominal ultrasound sonography test (USG) was performed, which revealed the following results: A region of mixed echogenicity measuring approximately 7.1 x 9.2 cm along the head of the pancreas and surrounded by fat stranding is most likely indicative of necrotizing focal pancreatitis. In response to the findings of USG, a high-resolution computed tomography (HRCT) of the abdomen was performed the following day, which revealed a large, well-oriented fluid density lesion measuring approximately 6x5x4 cm in the head of the pancreas, as well as calcification and a poorly defined mass surrounding the head of the pancreas (Figure 1).

Based on the findings of the CT scan, an endoscopic ultrasound (EUS) was conducted, revealing a large, solid, well-defined cystic mass with septations and vascularity measuring 2.5 x 3.3 cm in the head and the body of the pancreas. Another mass component in the head of the pancreas was hypoechoic, with calcified areas measuring 2 x 3 x 2.6 cm. The radiological evidence and clinical symptoms of the patient strongly suggested the presence of a localized pancreatic neuroendocrine tumor. Hence, for further grading and determining the course of treatment of the patient, a EUS-FNA for cytology, and a pancreatic head biopsy were performed for histopathology.

**Figure 2:**
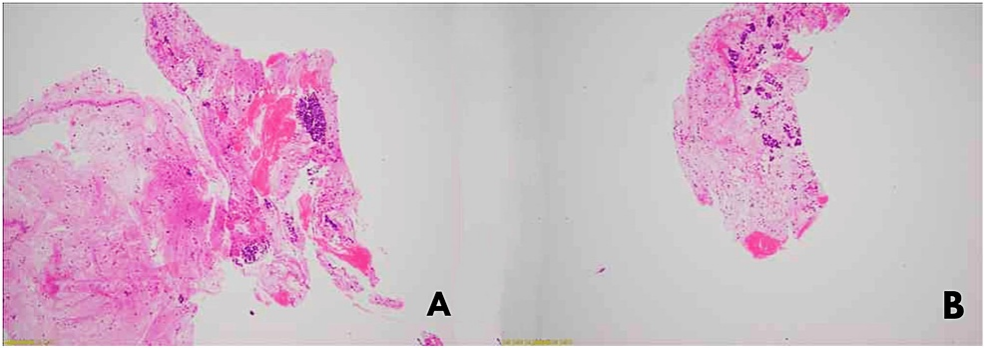
Histological examination of sections A & B reveals tissue cores showing a neoplasm arranged in nests on H&E stain

Histopathology confirmed the presence of a neuroendocrine tumor (NET) in the pancreatic head, revealing a nesting neoplasm with round-to-oval nuclei, salt-and-pepper chromatin, scant cytoplasm, and inconspicuous nucleoli in the individual tumor cells (Figure 2).

No areas of necrosis were identified. Immunostaining revealed cytokeratin and synaptophysin positivity with a Ki-67 index between 2% and 20%. His CA-19.9 levels were 4.99 U/ml (0-37 U/ ml). A conclusive diagnosis of grade-2 PNET was made based on histopathology and immunostaining. Given the state of his localized, well-differentiated, non-functional, grade-2 PNET and the lack of signs of metastatic or lymph node involvement, the decision to perform pancreatic sparing resection (central pancreatectomy and enucleation) was made. The patient was operated on successfully without any intraoperative or postoperative complications. Postoperative surveillance was done every three months for more than a year and no evidence of recurrence was found.

## Discussion

Rarely, the gastrointestinal sites, including the pancreas, liver, duodenum, and small intestine, can be affected by PNETs. In rare instances, it is impossible to pinpoint the genesis of a tumor [4]. Pancreatic neuroendocrine tumors are uncommon neuroendocrine neoplasms with a reported frequency of one per 100,000 and represent 1-2% of all pancreatic neoplasms [5]. There are no demonstrated differences in the epidemiology of PNETs based on race, gender, or geographic location [6]. The incidence, however, rises with age and peaks in the sixth and seventh decades [7].

The World Health Organization (WHO) classification method requires the evaluation of tumor localization, extension, proliferative potential, and angio/perineurial invasion. Well-differentiated endocrine tumors (WDET), endocrine carcinomas (WDEC), and poorly differentiated endocrine carcinomas (PDEC) are all categorized differently using this grading system [8]. For neuroendocrine tumors of the foregut, the European Neuroendocrine Tumor Society has suggested a TNM (tumor, node, metastasis) staging method with a grading system based only on the tumor's proliferative potential as measured by mitotic count and/or Ki-67 index [9]. However, Bilimoria et al. stated that the parameters linked with long-term survival following the excision of PNETs continue to be questioned [10]. NETs are typically tiny masses (<2 cm) at the time of diagnosis [4]; hence, symptoms caused by the compression of the tumor on adjacent organs are variable. According to hormone production, PNETs are classed as functional or non-functional depending on the existence or absence of symptoms [11]. These cancers have the ability to manufacture and release peptides and amino acids. When such compounds are released and activated, a clinical condition is produced. However, when these tumors emit inactive hormones or do not secrete them, they exhibit a condition with mass effect [3]. Unfortunately, in the majority of cases, nonspecific symptoms, such as stomach pain, may remain unexplained for months, and delayed care of the tumor can sometimes influence prognosis, particularly in the case of biologically aggressive NETs [4,12]. One percent (1%) to 2% of PNETs are related to family disorders, including type 1 multiple endocrine neoplasia, tuberous sclerosis, von Hippel-Lindau syndrome, and type 1 neurofibromatosis [13].

Multiple investigations on the pathogenesis of PNETs have revealed that chromosomal aberrations may play a role in the cause of PNETs. In the most recent WHO classification (2017), PNETs are classified based on their proliferative activity as evaluated by mitotic count and the expression of nuclear antigen Ki-67, a marker of cellular proliferation [7,14]. Non-functional pancreatic neuroendocrine tumors (NF-PNETs) continue to be uncommon entities. They are a heterogeneous group of cancers, and their identification is frequently delayed due to the absence of any hormonal symptoms [7,8]. The majority of symptoms, including stomach pain, weight loss, and abdominal mass, are caused by the mass effect. Nonetheless, NF-PNETs are increasingly discovered inadvertently during imaging [15].

Generally, the clinical presentation is determined by tumor mass effects. Therefore, conventional imaging methods often achieve localization [8]. Existing imaging methods include ultrasound, somatostatin receptor scintigraphy, computed tomography, positron emission tomography (PET), magnetic resonance imaging (MRI), and endoscopic ultrasound (EUS). These approaches may be beneficial for finding the majority of PNETs with a radius of 2 cm or more but are typically not helpful in PNETs less than 5 mm in diameter [16]. On computed tomography (CT), these lesions appear as circumscribed hypervascular solid masses that seldom restrict the pancreatic duct [17]. EUS is an outstanding modality that has been shown to be a valuable tool for the detection of PNETs, particularly in small tumors that cannot be detected by CT or MRI. Additionally, EUS has the added benefit of acquiring diagnostic biopsies [7]. Tumor indicators are helpful in the diagnosis, prognosis, therapy evaluation, and detection of NET recurrence. Nonspecific NET tumor markers, including chromogranin A (CgA), pancreatic polypeptide, and serum neuron-specific enolase have been utilized in patients with PNETs. CgA is regarded as the best overall neuroendocrine tumor marker and is elevated in 50-100% of patients with different NETs [18]. CgA has a sensitivity of 73% and a specificity of 95% in detecting NETs. Serum or plasma CgA levels reflect the tumor burden and are linked with disease progression. However, nonhormonal tumor biomarkers (e.g., CgA) are not necessary to be tested for patients with NF-PNETs [7].

Surgical resection is often the cornerstone of curative treatment. Curative surgery is advised for sporadic NF-PNETs if local and hepatic resectability is possible and extrahepatic metastases are absent. While 2 cm tumors may be amenable to enucleation, bigger masses necessitate extensive oncological excision [19]. PNETs do not typically respond well to chemotherapy and targeted medicines, and surgical excision with a curative aim is the treatment of choice [7]. NF-PNETs have a dismal prognosis compared to pancreatic adenocarcinoma and functional PNETs, with a five-year survival rate of 60%-100% for localized disease, 40% for regional, and 29% for distant metastases [20]. Age >55 years, NF-PNET, poor tumor differentiation, distant metastases, and surgical method were identified as characteristics that negatively influenced survival. In the case of NF-PNETs, five-year survival rates range between 26% and 58% [8].

## Conclusions

Non-functional PNETs are a rare kind of cancer. Unlike the functional types of PNETs, they do not produce hormonal products and hence they are difficult to diagnose. The ultrasound of the abdomen is the initial test of choice, which could be followed by investigations with higher sensitivity and specificity. Once diagnosed, resection of the tumor should be done and the patients should be followed up for recurrences.
